# Single-Cell Transcriptomic Analysis Reveals the Crosstalk Propensity Between the Tumor Intermediate State and the CD8+ T Exhausted State to be Associated with Clinical Benefits in Melanoma

**DOI:** 10.3389/fimmu.2022.766852

**Published:** 2022-07-12

**Authors:** Jiali Zhu, Min Yan, Haoteng Yan, Liwen Xu, Zedong Jiang, Gaoming Liao, Yao Zhou, Wei Liu, Xin Liang, Xia Li, Yun Xiao, Yunpeng Zhang

**Affiliations:** ^1^ College of Bioinformatics Science and Technology, Harbin Medical University, Harbin, China; ^2^ Key Laboratory of High Throughput Omics Big Data for Cold Region’s Major Diseases in Heilongjiang Province, Harbin Medical University, Harbin, China

**Keywords:** cell-cell communication, cell state, melanoma, single-cell transcriptome analysis, CD8+ T cell

## Abstract

Heterogeneous crosstalk between tumor cells and CD8+ T cells leads to substantial variation in clinical benefits from immunotherapy in melanoma. Due to spatial distribution and functional state heterogeneity, it is still unknown whether there is a crosstalk propensity between tumor cells and CD8+ T cells in melanoma, and how this crosstalk propensity affects the clinical outcome of patients. Using public single-cell transcriptome data, extensive heterogeneous functional states and ligand–receptor interactions of tumor cells and CD8+ T cells were revealed in melanoma. Furthermore, based on the association between cell–cell communication intensity and cell state activity in a single cell, we identified a crosstalk propensity between the tumor intermediate state and the CD8+ T exhausted state. This crosstalk propensity was further verified by pseudo-spatial proximity, spatial co-location, and the intra/intercellular signal transduction network. At the sample level, the tumor intermediate state and the CD8+ T exhausted state synergistically indicated better prognosis and both reduced in immunotherapy-resistant samples. The risk groups defined based on these two cell states could comprehensively reflect tumor genomic mutations and anti-tumor immunity information. The low-risk group had a higher *BRAF* mutation fraction as well as stronger antitumor immune response. Our findings highlighted the crosstalk propensity between the tumor intermediate state and the CD8+ T exhausted state, which may serve as a reference to guide the development of diagnostic biomarkers for risk stratification and therapeutic targets for new therapeutic strategies.

## Introduction

Skin cutaneous melanoma (SKCM) is the most lethal malignancy of the skin and is characterized by high inter-tumor and intra-tumor heterogeneity due to its high mutational load and increased cellular plasticity (1–3). Recently, numerous researchers have demonstrated the existence of phenotypic and transcriptional subpopulations of tumor cells (3, 4) and CD8+ T cells (4–6) in melanoma, often referred to as cell states, both within and between patients. The *MITF* (melanocyte-inducing transcription factor)-rheostat model incorporates the six different phenotypic states found in melanoma to date. These cell states are ranked from high to low according to *MITF* activity into the hyperdifferentiated state, the melanocytic state, the intermediate state, the starvation state, the neural crest stem-like cell state, and the mesenchymal-like state (7). Intratumor CD8+ T-cell population showed strong similarity in three independent melanoma cohorts, with greater heterogeneity in subsets of CD8+ T cells, including transitional and exhausted states, compared with naive/memory and cytotoxic CD8+ T cells (4, 5, 8). The analysis of the heterogeneity of cell states makes it crucial to explore the mechanisms generating the different cell states and how individual cell states influence melanoma biological processes, such as metastasis (3) and therapy response (5, 9–13). The single-cell analysis of CD8+ T cells revealed that TCF7+ memory-like state frequency in tumor tissue predicts immunotherapy response and better survival (5).

Technological advances have allowed single-cell analysis to reveal that cell–cell communication (CCC) plays a crucial role in numerous biological processes by a dynamic communicating network formed through communication and cooperation between cells, such as tissue homeostasis (14, 15), cell development (16–18), disease pathogenesis and progression (4, 19), and therapy resistance (20). CCC is ubiquitous in melanoma ecosystems composed of multiple cell types and is often mediated by ligand–receptor interactions (4, 19). Moreover, accumulated evidence demonstrated that CCC between tumor cells and CD8+ T cells can influence cellular functions. For instance, *PD-L1* (programmed cell death 1 ligand 1)–*PDCD 1* (programmed cell death 1) (21, 22), *CD38* molecule–adenosine receptor (23), and loss of HLA related ligand–receptor interactions (24) can suppress the effector function of CD8+ T cells. Among CCC between tumor cells and CD8+ T cells, some ligands or receptors are cell state-specific; for example, *PD-L1* is a classical marker of the CD8+ T exhausted state (25). In addition to ligand–receptor interactions, the distance between immune cells and tumor cells might directly reflect the lethality of immune cells toward tumors or, *vice versa*, the interference of tumor cells with immune cells (26). The spatial distribution analysis demonstrated that *PD-L1*+ cells within proximity to tumor cells and intra-tumoral CD8+ density predict response to anti-PDCD1 therapy in melanoma (27).

In the present study, we utilized the public single-cell transcriptome data to explore the crosstalk propensity between tumor cells and CD8+ T cells based on the association between CCC and cell state in melanoma. The crosstalk propensity between the tumor intermediate state and the CD8+ T exhausted state was identified and verified, where those two cell states were both associated with better prognosis and reduced in immunotherapy-resistant samples. Our goal was to explore the underlying mechanisms associated with tumor progression and immunotherapy response from the crosstalk propensity perspective.

## Materials and methods

### Data Collection of Melanoma Samples

The processed scRNA-seq dataset was downloaded from the GEO database under the accession code GSE115978, where tumor cells and CD8+ T cells were extracted according to the cell labels defined in the original studies. Raw read counts were counts per million (CPM)-normalized and genes that were expressed in less than 10% of the CD8+ T and tumor cells were filtered out using Seurat4.0 R package, respectively (28). The gene expression data, mutation data, clinical data, and immune feature profiles of the TCGA-SKCM cohort were available in the article (29). Bulk expression profiles, namely, GSE22153 and GSE91061, were also obtained for survival analysis and immunotherapy resistance analysis, respectively. The bulk datasets for primary and metastatic analyses were gathered from the GEO database under the accession codes GSE8401, GSE46517, and GSE59455. The mRNA profiles of 48 melanoma cell lines before and after 6 hours of treatment with interferon-gamma were downloaded under the accession code GSE154996. The RNA-seq data of CD8+ tumor-infiltrating lymphocytes from wild-type and *Prdm1* conditional knockout (cKO) mice bearing B16F10 melanoma were obtained by GSE113221. Moreover, spatial transcriptome data and H&E-stained annotation information of a melanoma sample were obtained from previous research (30). The RNA-seq data of 222 histologically distinct micro-regions (~5–20 cells per region) extracted from a melanoma patient were downloaded under the accession code GSE171888.

### Inference of Tumor Cell States

The defined transcriptional factor (TF) motif-based regulons related to tumor cell states were collected from the published research (3) (**Table S1**). Among those regulons, we applied the AUCell method to calculate activities of regulons with normalized count profile using the AUCell R package (31) and scaled activities by the maximum difference normalization method. Consensus unsupervised clustering result was obtained based on 1,000 k-means clustering of scaled regulons activities using the ComplexHeatmap R package (32). The clustering results with K set to 4 best matched the TF regulons’ pattern of tumor cell states reported in previous research (3). In addition, we used the gene signatures obtained from the literature (3) and CancerSEA (33) to characterize and validate the functional features of identified tumor clusters (**Table S1**).

### Inference of CD8+ T-Cell States

The top 2,000 variable genes were identified from the normalized and scaled data, and principal component analysis (PCA) was performed on the expression matrix of the variable genes. Clusters were detected through the FindClusters function at resolution 0.5 and visualized by uniform manifold approximation and projection (UMAP). Then, CD8+ T clusters were annotated using canonical cell state markers (25) (**Table S1**) and cluster-specific markers identified by the FindAllMarkers function (log fold change threshold of 0.25 and FDR threshold of 0.05). Meanwhile, the top 2,000 variable genes were also used as input to the Monocle 2 algorithm (34) to construct the development trajectory of diverse CD8+ T-cell subpopulations.

### Identifying Significant Ligand–Receptor Interactions

We integrated known ligand–receptor pairs from five public resources and screened 3,218 pairs supported by at least two resources for subsequent analysis (35–39) (**Figure S1A**, **Table S2**). We then defined a ligand or receptor as an “expressed” gene in a certain cell cluster if more than 20% of cells had its expression level by cutoff 0, and set the expression value of unexpressed ligand/receptor to zero. The interaction score of a given ligand–receptor pair between cell cluster A and cell cluster B was the product of average ligand expression across all cells in cluster A and the average receptor expression across all cells in cluster B. Statistical significance was then assessed by randomly shuffling the cell state labels of all tumor cells and CD8+ T cells respectively and repeating the above steps, which generated a null distribution of interaction score for each ligand–receptor pair in each pairwise comparison between tumor clusters and CD8+ T clusters. After running 1,000 times permutations, *p*-values were calculated as the fraction of permuted ligand–receptor interaction scores larger than real interaction scores.

The ligand–receptor interaction network was visualized by Cytoscape, and functional enrichment analysis and gene mode analysis in the protein–protein interaction network were performed by Metascape at http://metascape.org/gp/index.html#/main/step1 (40).

### Identifying the Crosstalk Propensity Between Tumor Cells and CD8+ T Cells

To better correlate CCC with cell state at the single-cell level, we applied NicheNet to predict ligand activities in each signal receiver cell by the predict_ligand_activities function in default parameters (41). The differential gene signature was identified as cell state-specific signatures through the FindAllMarkers function in Seurat (log fold change threshold of 0.25 and FDR threshold of 0.05).

Next, we identified cell state-related ligands during signal transduction. For sender cells, we identified cell state-related ligands whose expression levels were significantly correlated with the average expression of cell state-specific signature (Pearson correlation test, FDR < 0.05). For receiver cells, we identified state-related ligands whose predicted activities were significantly correlated with the average expression of cell state-specific signature (Pearson correlation test, FDR < 0.05). The above analyses were performed in parallel with the two signal transduction directions (from CD8+ T to tumor and from tumor to CD8+ T). During signal transduction, the ligands both significantly related to a tumor state and a CD8+ T state were considered as the shared ligands between them. The hypergeometric test analysis was performed to explore whether the number of shared ligands has a significant over-occurrence, which could indicate a significant association between CCC and cell states.

In addition, we repeated the above analysis when CD8+ T cells act as senders, replacing the cell state-specific signatures of tumor cells and CD8+ T cells with tumor cell state-related TF sets and the canonical cell function markers of the CD8+ T cell, respectively.

### Pseudo-Space Construction

Using cell state labels and TPM expression profile of tumor cells and CD8+ T cells as input, three-dimensional pseudo space analysis was carried out by CSOmap (39) at https://doi.org/10.24433/CO.8641073.v1.

### The Inter/Intra-Cellular Signal Network Construction

First, we applied scMLnet (42) to obtain intercellular ligand–receptor interactions and intracellular signal transduction networks in signal receiver cells and constructed ligand–receptor–TF links. Then, transcriptional regulons were identified using SCENIC (31) to obtain candidate TF–ligand links. Finally, we integrated the above results by connecting TF–ligand links and ligand–receptor–TF links with the intersection of ligands as the intermediary to construct the TF–ligand–receptor–TF network.

### Calculation of Cell State Abundance Score

For spatial transcriptome data and bulk transcriptome data TCGA-SKCM, GSE22153, GSE91061, GSE8401, GSE46517, GSE59455, GSE154996, GSE171888, and GSE113221, single-sample gene set enrichment analysis (ssGSEA) (43) was applied to calculate activity scores for the tumor intermediate state and the CD8+ T exhausted state based on cell state-specific signatures. The ssGSEA algorithm was implemented in the GSVA package.

### Survival Analysis

Hazard ratios (HRs) and *p*-values were derived using a Cox proportional hazards model to evaluate the prognostic effect of ssGSEA score of cell state-specific signature in both TCGA-SKCM and GSE22153 cohorts. Patients in cohorts were classified into two groups according to the median value of the ssGSEA scores. Kaplan–Meier survival curves were used to visualize survival differences between the two groups, and log-rank test was utilized to assess the significance.

### Risk Group Analysis

According to the median values of ssGSEA scores of the tumor intermediate state and the CD8+ T exhausted state, the patients in the TCGA-SKCM cohort were classified into high- and low-risk groups. Patients in the low-risk group not only had higher ssGSEA scores of the tumor intermediate state but also had higher ssGSEA scores of the CD8+ T exhausted state. The other patients were in the high-risk group. Fisher’s test was used to explore the association between risk groups and other features of patients, including immune subtypes, mutant subtypes, and gene mutations.

### Statistical Analysis

Kaplan–Meier curves and forest plot were visualized using the survminer package. The HRs and *p*-value were calculated with survival package. The significance of differences between the two groups was determined by Wilcoxon rank-sum test. The chi-squared test and Fisher’s test were used to determine the significance of the overlap between two categorical variables. Pearson correlation test was used to explore the correlation between two continuous variables. All analyses were performed in R version 4.0.5. “ns” denoted non-significant, * denoted *p* < 0.05, ** denoted *p* < 0.01, *** denoted *p* < 0.001, and **** denoted *p* < 0.0001.

## Results

### Inference Cell States of Tumor Cells and CD8+ T Cells

**Figure 1:**
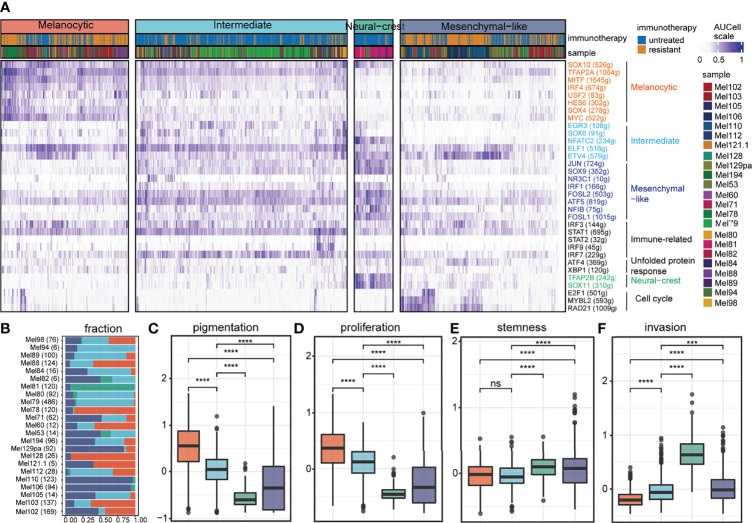
Inferred cell states of tumor cells from SKCM patients. **(A)** Unsupervised clustering results of 2,018 tumor cells (samples *n* = 23) based on regulon activity profiles, including 4 clusters annotated by different colors. For each TF (rows), the number of genes contained in its regulon is shown in parentheses and its cell state specificity is indicated by color. **(B)** The composition of tumor cell states in each sample, where the number of cells contained in the sample is shown in parentheses on each row. **(C-F)** The functional differences between the 4 tumor states are shown in the box diagram, including pigmentation shown in **(C)**, proliferation shown in **(D)**, stemness shown in **(E)**, and invasion shown in **(F)**. ns denoted non-significant, *** denoted p < 0.001, and **** denoted p < 0.0001.

According to transcriptional regulons related to tumor cell states in previous research (3), four cell states were identified by unsupervised clustering analysis on the regulon activity profile of 2,018 tumor cells (**Figure 1A**; gene names are listed in **Table S1**). Tumor cell states could coexist in melanoma patients rather than just one tumor cell state (**Figure 1B**); this phenomenon has been reported in multiple studies (4, 13). The melanocytic state exhibited elevated regulon activities of lineage-specific transcription factors (e.g., *SOX10* and *MITF*) and significantly higher pigmentation and proliferation potential (**Figures 1A,C,D**). Compared to the melanocytic state, the mesenchymal-like state lost regulon activities of melanocytic transcription factors and had significantly higher stemness and invasion potential (**Figures 1A,E,F**). The majority of the tumor cells were in the intermediate state, which was governed by the *EGR3*, *NFATC2*, and *SOX6* (**Figure 1A**). The intermediate state was also characterized by intermediate *MITF* regulon activity and increased regulon activities related to mesenchymal-like (e.g., *JUN* and *SOX9*) and immune modulators (e.g., *IRF3* and *STAT1*), which was a transitional state from a melanocytic state to a mesenchymal-like state as previous research reported (3) (**Figure 1A**). A few tumor cells resided in a neural crest-like state, as identified by co-localized *SOX11* and *TFAP2B* regulon activities, and exhibited a low level of melanocytic regulon activity compared to intermediate, except for *SOX10* (**Figure 1A**).

**Figure 2:**
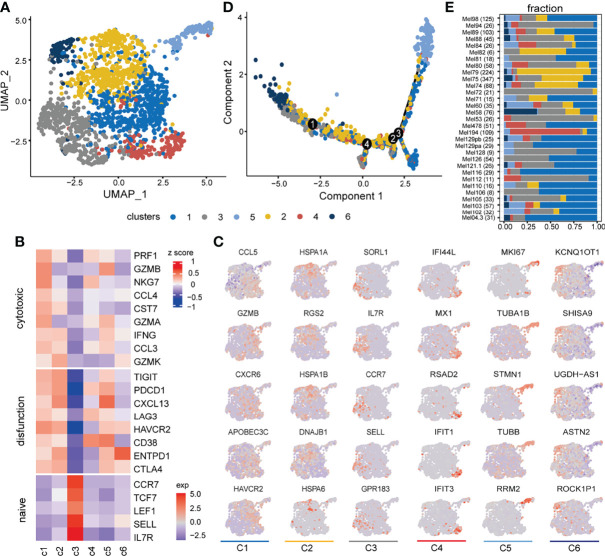
Inferred cell states of CD8+ T cells from SKCM patients. **(A)** UMAP plot of 1,759 CD8+ T cells (samples *n* = 31), including 6 clusters annotated by different colors. **(B)** Heatmap showing expression levels of canonical CD8+ T-cell function-associated markers in each CD8+ T cluster. **(C)** UMAP feature plot representation of selected cluster-specific differential markers within individual CD8+ T-cell clusters. Each column corresponds to six clusters in order. **(D)** The trajectory distribution of CD8+ T-cell clusters. **(E)** The composition of CD8+ T-cell clusters in each sample, where the number of cells contained in the sample is shown in parentheses on each row.

With graph-based clustering on the expression profile of 1,759 CD8+ T cells, six clusters were detected (**Figure 2A**). The expression levels of well-known functional state markers (25) and cluster differential genes suggested cell states of CD8+ T cells (**Figures 2B,C**; gene names are listed in **Table S1**). Cluster C1 was denoted as exhausted state, which exhibited the elevated expression of exhausted markers (e.g., *HAVCR2*, *PDCD1*, and *LAG3*) and cytotoxicity-related genes (e.g., *GZMB*, *PRF1*, *GZMA*, and *NKG7*) (4, 8). Cluster C2 was defined as a transitional state as it moderately expressed cytotoxic markers and highly expressed *GZMK*, which widely featured the intermediate state between naive and exhausted T-cell states (8). Cluster C3 resembled the naive-like CD8+ T state through the enrichment of naive/memory-related genes (e.g., *IL7R*, *CCR7*, *SELL*, and *TCF7*). The state definition of the above three major cell clusters was consistent with a continuous progression process in trajectory analysis (**Figure 2D**). In addition, there were three other smaller clusters, in which cluster C4 expressed a higher level of interferon induction gene (e.g., *IFI44L*, *IFIT1*, and *IFIT3*). Compared to C2, C5 expressed a low level of *GZMK* and additional proliferation gene *MKI67*. Cluster C6 expressed high-level *ENTPD1* and was located at the beginning of the trajectory and close to naive-like cells (**Figure 2D**). In addition, the above CD8+ T-cell subsets have varying proportions from each patient (**Figure 2E**).

### The Landscape of Tumor–CD8+ T Crosstalk Characterized by Ligand–Receptor Interactions

Here, the scRNA-seq dataset was annotated by integrated human ligand–receptor pairs from five published resources (35–39) (**Figure S1A**, gene names are listed in **Table S2**). These annotations were used to infer putative cell state-specific ligand–receptor interactions to construct a tumor–CD8+ T interactome (19, 39) (**Figures S1B, C**; for details, see *Materials and Methods*), resulting in a ligand–receptor interaction network (**Figure S2A**). Genes in this network were involved in cell adhesion, leukocyte migration, extracellular matrix organization, and immune response (**Figures S2B-D**).

**Figure 3:**
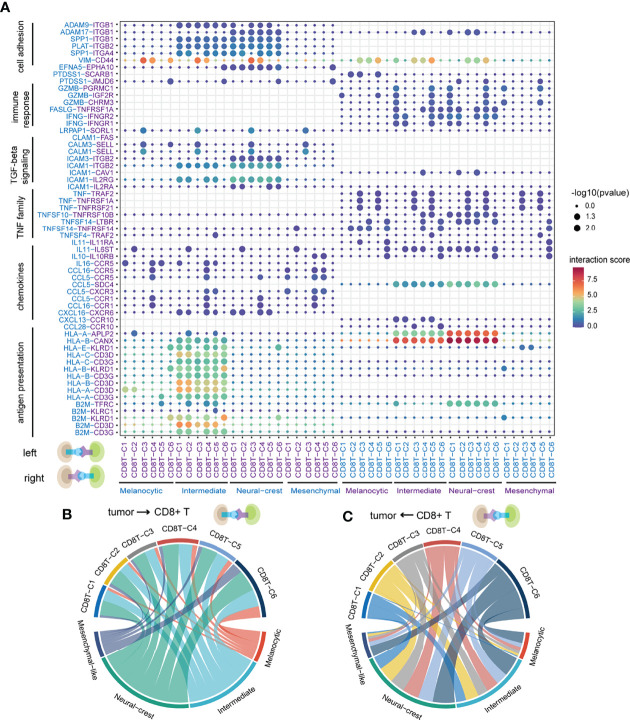
The landscape of ligand–receptor interactions between tumor cells and CD8+ T cells. **(A)** Bubble heatmap showing the interaction strength for selected ligand–receptor pairs. Dot size indicates *p*-value generated by the permutation test and dot color represents interaction strength. **(B)** The number of significant ligand–receptor pairs between tumor cells and CD8+ T cells when tumor cells act as senders. Each link is colored by the tumor cell states and link thickness represents the number of significant ligand–receptor pairs. **(C)** The number of significant ligand–receptor pairs between tumor cells and CD8+ T cells when CD8+ T cells act as senders. Each link is colored by the CD8+ T-cell states.

Notably, tumor cells expressed relatively high levels of chemokines and ligands associated with antigen presentation and TGF-beta signaling pathway, while the corresponding receptors were widely expressed in CD8+ T cells, suggesting that these ligands play significant roles in influencing immune cell infiltration in melanoma (**Figure 3A**). Conversely, ligand *IFNG* and its related pro­inflammatory cytokine *TNF*, and ligands related to tumor necrosis factor family and cytotoxicity were expressed in CD8+ T cells, indicating the killing potential of CD8+ T cells against tumor cells (**Figure 3A**). Interactions of *VIM*-*CD44* between tumor cells and CD8+ T cells were commonly observed, which was involved in epithelial–mesenchymal transition (44, 45).

Of note, intermediate and neural-crest-like tumor cells have the highest number of and largely shared ligand–receptor interactions out of all cell states, whether tumor cells act as senders or receivers (**Figures 3B,C**, **S1D**). Those results suggested that intermediate and neural-crest-like tumor cells may more frequently crosstalk with CD8+ T cells. Specifically, ligand–receptor interactions related to antigen presentation machinery (e.g., *HLA-A*-*CD3G* and *B2M*-*KLRD1*) were mainly observed between intermediate tumor cells and almost all CD8+ T clusters (**Figure 3A**). The above findings implied that intermediate tumor cells are capable of presenting more immunogenicity. CD8+ T clusters C1 and C5 expressed *GZMB* interacting with the corresponding receptors that were expressed in intermediate and neural-crest-like tumor cells, suggesting the presence of cytotoxic effect in the two tumor cell states (46) (**Figure 3A**). In particular, *PTDSS1-SCARB1* was only observed between CD8+ T cells and melanocytic tumor cells (**Figure 3A**). The loss of *SCARB1* has been demonstrated to downregulate TF *MITF* related to the melanocytic state in human melanoma (47), suggesting that ligand–receptor interaction may affect the cell state of signal receivers.

### Deciphering the Crosstalk Propensity Between Tumor Cells and CD8+ T Cells

**Figure 4:**
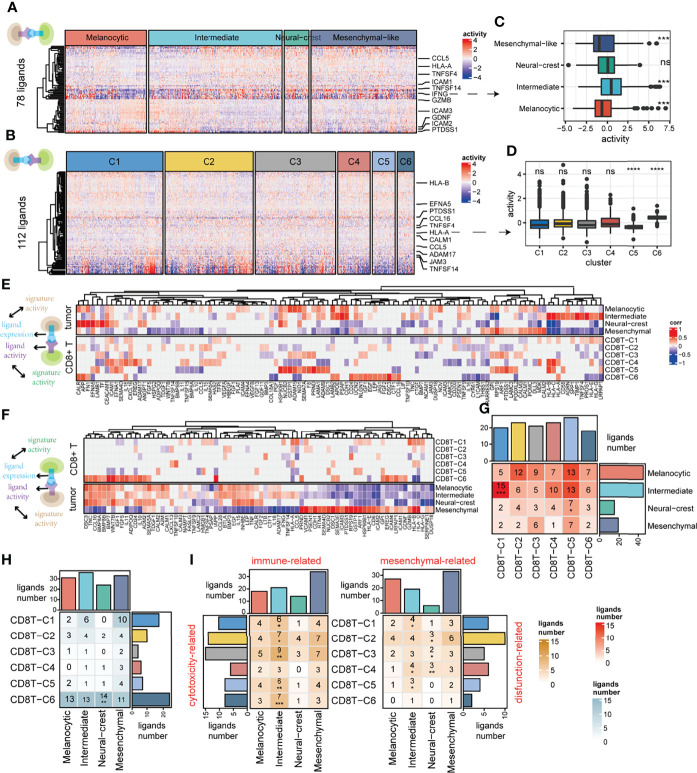
Deciphering the association between cell–cell communication and cell state. **(A)** Among ligands expressed by CD8+ T cells (*n* = 78), predicted ligand activity in receiver tumor cells by the NicheNet method. **(B)** Among ligands expressed by tumor cells (*n* = 112), predicted ligand activity in receiver CD8+ T cells by the NicheNet method. **(C)** The difference in predicted ligand *IFNG* activities between tumor cell states. **(D)** The difference in predicted *HLA-A* activities between CD8+ T clusters. **(E)** The top panel of the correlation heatmap shows the ligands associated with the tumor cell states, and the bottom panel shows the ligands associated with the CD8+ T-cell states when CD8+ T cells act as signal receivers. **(F)** The top panel of the correlation heatmap shows the ligands associated with the CD8+ T-cell states, and the bottom panel shows the ligands associated with the tumor cell state when the tumor cells act as signal receivers. **(G, H)** The intersection of cell state-related ligands between tumor cells and CD8+ T cells when CD8+ T cells act as signal receivers is shown in **(G)**, as well as when tumor cells act as signal receivers shown in **(H)**. **(I)** The intersection of function-related ligands between tumor and CD8+ T cells when tumor cells act as signal receivers. ns, non-significant, * denoted p <0.05, ** denoted p <0.01, *** denoted p <0.001, and **** denoted p <0.0001.

To further identify ligand–receptor interactions predicted to be associated with cell states, our analysis firstly applied NicheNet (41) to obtain the ligand activity of each signal receiver cell (**Figures 4A–B**). Obvious differential activities of some ligands between tumor cell states were observed when tumor cells act as receivers, such as intermediate tumor cells that received the strongest ligand *IFNG* signal compared to other tumor cell states (**Figures 4A,C**). However, this phenomenon was not obvious when CD8+ T cells act as receivers (**Figures 4B, D**). Next, we identified cell state-related ligands when cells act as senders (**Figures 4E,F**, top) and receivers (**Figures 4E,F**, bottom) (for details, see *Materials and Methods*; Pearson correlation test, FDR < 0.05). Interestingly, there were very few CD8+ T-cell state-related ligands when they act as senders, it is speculated that CD8+ T cells may use the common ligands to perform some functions together (**Figure 4F**, top).

We further explore the association between CCC and cell state using cell state-related ligands as mediators (for details, see *Materials and Methods*). The significant over-occurrence of ligands shared between the tumor intermediate state and the CD8+ T exhausted state was observed when tumor cells send signals to CD8+ T cells (hypergeometric test, *p* < 0.001), which figured out that intermediate tumor cells may affect CD8+ T exhausted state cells by those shared ligands (**Figure 4G**). However, this phenomenon was not detected in the other case when CD8+ T cells act as sender cells (**Figure 4H**). To further verify whether the above phenomenon is due to CD8+ T cells functioning through common ligands rather than state-specific ligands (**Figure 4F**, top), we repeated this process with functionally dependent ligands, such as cytotoxic, exhausted, and naive ones (for details, see *Materials and Methods*). Indeed, the cytotoxicity-related ligands in almost all CD8+ T cells significantly cooccurred with immune-related ligands in intermediate tumor cells (**Figure 4I**, left). In addition, exhaustion-related ligands in CD8+ T exhausted cells significantly cooccurred with mesenchymal-related ligands in intermediate tumor cells (**Figure 4I**, right). In general, the influence of ligand–receptor interaction between tumor and CD8+ T cells is more likely to occur between the CD8+ T exhausted state and the tumor intermediate state, suggesting a crosstalk propensity between them.

### Verifying the Crosstalk Propensity Between the Tumor Intermediate State and the CD8+ T Exhausted State

**Figure 5:**
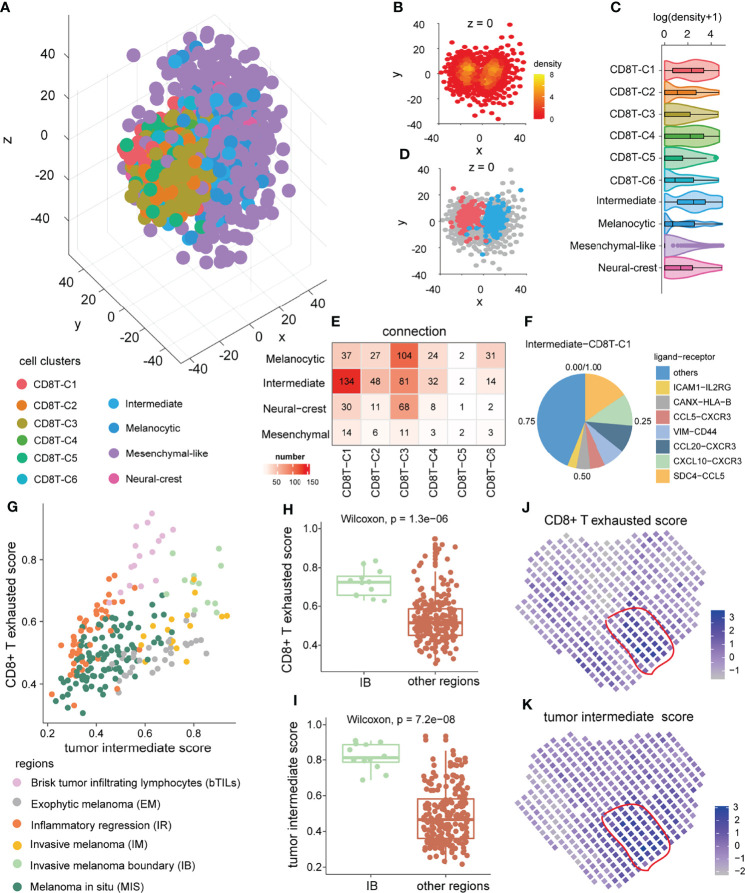
Pseudo-space proximity and spatial co-location between the tumor intermediate state and the CD8+ T exhausted state. **(A)** Spatial organization of tumor cells and CD8+ T cells in the pseudo-space inferred by CSOmap based on the scRNA-seq data. Each dot represents a cell, and its color represents the corresponding cell state. **(B)** The cross-section of *z* = 0 of the pseudo-space. The color of the dots represents cell density. **(C)** The difference in cell density between 10 cell clusters of CD8+ T cells and tumor cells. **(D)** Location of intermediate tumor cells (blue) and CD8+ T exhausted cells (red) in the cross-section of pseudo-space *z* = 0. **(E)** Numbers of cell–cell connections between tumor cell states and CD8+ T-cell states. **(F)** The top ligand–receptor interactions and their contribution to pseudo-space proximity of the tumor intermediate state and the CD8+ T exhausted state. **(G)** The distribution of ssGSEA score of the tumor intermediate state and the CD8+ T exhausted state in distinct annotated microregions from melanoma tumor MEL1-1 (GSE171888). **(H, I)** The difference in tumor intermediate state ssGSEA score **(H)** and CD8+ T exhausted state ssGSEA score **(I)** of micro-regions between invasive melanoma boundary region and other regions in the melanoma tumor MEL1-1. **(J, K)** The CD8+ T exhausted state ssGSEA score **(J)**, and tumor intermediate state ssGSEA score **(K)** of spatial spots in another melanoma sample (30).

Considering that cell function is often influenced by neighboring cells (48), we subsequently verified this crosstalk propensity from the perspective of space. The three-dimensional pseudo space was reconstructed by the CSOmap algorithm (39) based on single-cell expression profiles of tumor cells and CD8+ T cells (**Figure 5A**). In pseudo space, CD8+ T exhausted cells and intermediate tumor cells formed the major part of tightly linked structures (**Figures 5B,C**) and were closed to each other at the boundary between the tumor cells and CD8+ T cells (**Figure 5D**). Quantitatively, the tumor intermediate state and the CD8+ T exhausted state had the highest overall connected cell pairs (**Figure 5E**), indicating their pseudo-spatial proximity. The pseudo-space proximity was mainly contributed by antigen presentation and chemokine-related ligand–receptor interactions, such as *HLA-B*-*CANX* and *CXCL10-CXCR3* (**Figure 5F**). Interestingly, in the microregion sequencing data of a melanoma tumor MEL1-1 (49), the significantly increased ssGSEA scores of the CD8+ T exhausted state as well as the tumor intermediate state were found in the invasive melanoma boundary (IB) region (**Figures 5G-I**). In addition, in the spatial transcriptome data of a melanoma sample (30), some spatial spots were found to enrich the ssGSEA scores of the tumor intermediate state and the CD8+ T exhausted state simultaneously (**Figures 5J,K**). Those results suggested their spatial co-localization.

**Figure 6:**
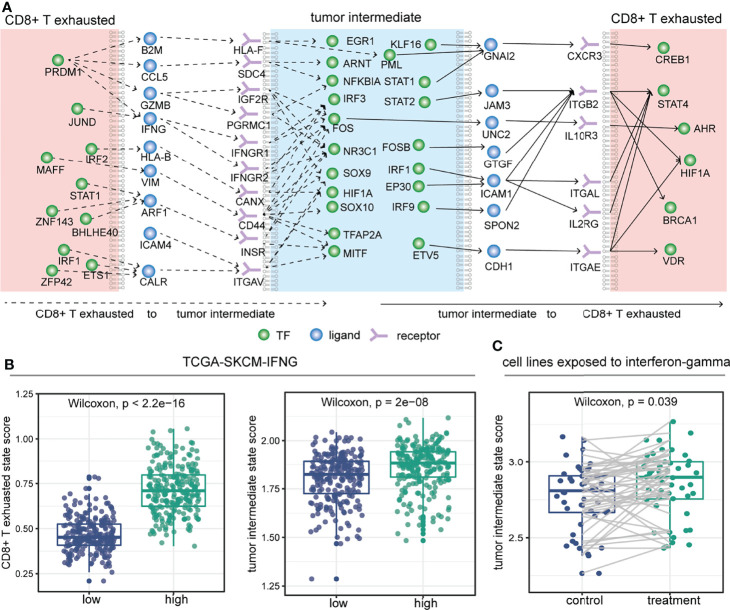
Inter/intracellular signal transduction network between the tumor intermediate state and the CD8+ T exhausted state. **(A)** Selected inter/intracellular signal transduction network (TF–ligand–receptor–TF) between the tumor intermediate state and the CD8+ T exhausted state. **(B)** Comparison of CD8+ T exhausted state ssGSEA score (left) and tumor intermediate state ssGSEA score (right) between high- and low-expressed groups defined by the median expression level of *IFNG* in the TCGA-SKCM cohort. **(C)** In 48 melanoma cell lines (GSE154996), comparison of tumor intermediate state ssGSEA score between before (control) and after 6 hours (treatment) of treatment with interferon-gamma.

To further investigate which molecules might mediate the crosstalk propensity between the tumor intermediate state and the CD8+ T exhausted state, we constructed an inter/intracellular signal transduction network (TF–ligand–receptor–TF) and found multiple signaling pathways involved in it (**Figures 6A** and **S3**). Importantly, some signal transduction links could further confirm the association between CCC and cell state. For instance, *PRDM1* could regulate the expression level of *IFNG* ligand that was secreted by CD8+ T exhausted cells and could bind to the *IFNGR1*/*IFNGR2* receptors on the intermediate tumor cells, and then activate the downstream mesenchymal-related TFs, such as *FOS* and *NR3C1*. The expression levels of those molecules in the above TF–ligand–receptor–TF link were almost significantly positively associated with the CD8+ T exhausted state and the tumor intermediate state (**Figures 6B**, **S4A,B**, Wilcoxon rank-sum test). To validate it, we collected experimental datasets and found that the expression level and ssGSEA score of the CD8+ T exhausted specific signature were reduced in CD8+ tumor-infiltrating lymphocytes of *Prdm1* cKO mice compared to wild type (**Figures S4C,D**). A recent study also demonstrated that *PRDM1* is essential for the differentiation of melanoma and its high expression level indicates better survival in melanoma (50). In particular, for *IFNG* ligand, interferon-gamma treatment could induce increased activity of the tumor intermediate state in 48 melanoma cell lines (**Figure 6C**, Wilcoxon rank-sum test). To some extent, these results reflected the influence of the TF–ligand–receptor–TF link on cell state, and these TF–ligand–receptor–TF link molecules may provide new insight into developing potential therapeutic targets. Collectively, the crosstalk propensity between the tumor intermediate state and the CD8+ T exhausted state was verified by three aspects: pseudo-space proximity, spatial co-localization, and signal transduction network.

### The Tumor Intermediate State and the CD8+ T Exhausted State are Synergistically Associated with Clinical Benefit

**Figure 7:**
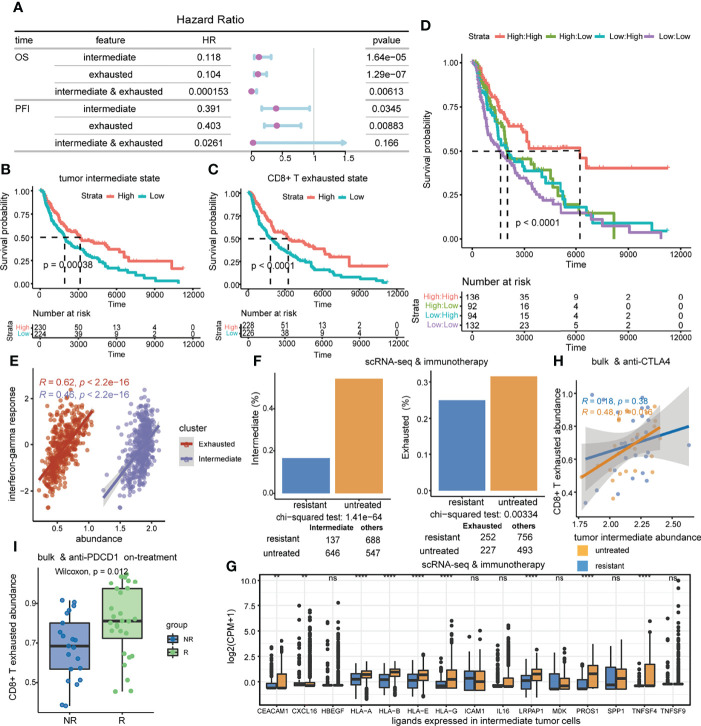
The association of the tumor intermediate state and the CD8+ T exhausted state with clinical benefit. **(A)** Prognostic value of tumor intermediate state and CD8+ T exhausted state ssGSEA scores in the TCGA-SKCM cohort. Forest plots show HRs (purple circle) and 95% confidence intervals (horizontal ranges) derived from Cox regression survival analyses for overall survival and progression-free interval. **(B–D)** Kaplan–Meier survival curves of overall survival by tumor intermediate state ssGSEA score **(B)**, CD8+ T exhausted state ssGSEA score **(C)**, and the combination of those two score variables **(D)** in the TCGA-SKCM cohort. **(E)** Correlations of interferon-gamma response scores with tumor intermediate state ssGSEA scores as well as CD8+ T exhausted state ssGSEA scores. **(F)** The difference in the proportion of intermediate tumor cells and exhausted CD8+ T cells between immunotherapy untreated and resistant samples in the scRNA-seq dataset. **(G)** The difference in the expression level of shared ligands in intermediate tumor cells between immunotherapy untreated and resistant samples in the scRNA-seq dataset. **(H)** The correlations of the tumor intermediate state with the CD8+ T exhausted state, which are compared between anti-CTLA4 untreated and progressed samples in the GSE91061 cohort. **(I)** Comparison of CD8+ T exhausted state activities between responder and non-responder patients who were on anti-PDCD1 treatment in the GSE91061 cohort.

Subsequently, we explored whether this crosstalk propensity may be used to gain predictive insights into relevant biological phenotypes of interest. Surprisingly, there was no difference in ssGSEA scores of the tumor intermediate state and the CD8+ T exhausted state between primary and metastatic patients in melanoma (**Figures S5A,B**). Therefore, the influence of primary and metastatic factors on the results was not considered in the following analysis. Cox proportional hazards (COX-PH) models and survival curve analysis revealed that the high ssGSEA score of the tumor intermediate state and the CD8+ T exhausted state both significantly predicted favorable overall survival (OS) in the TCGA-SKCM cohort (intermediate: HR = 0.118, coxph *p* = 1.64e-05, log-rank *p* = 0.00038; exhausted: HR = 0.104, coxph *p* = 1.29e-07, log-rank *p* < 0.0001) (**Figures 7A–C**), as well as progression-free interval (PFI) (intermediate: HR = 0.391, *p* = 0.0345; exhausted: HR = 0.403, *p* = 0.00883) (**Figure 7A**). Intriguingly, the interaction of the two state score variables was identified as a better indicator of OS than any of the individual variables by a multiplication term in the COX-PH model (HR = 0.000153, *p* = 0.00613), but it was not found in PFI (**Figure 7A**). Consistently, survival curve analysis also showed that patients with both higher tumor intermediate state and CD8+ T exhausted state scores had significantly longer OS compared to the other groups (log-rank *p* < 0.0001) (**Figure 7D**). The above findings were confirmed by an independent cohort (**Figures S5C-E**).

Furthermore, we analyzed the role of the tumor intermediate state and the CD8+ T exhausted state in immunotherapy response. Positive associations were significant between interferon-gamma response and ssGSEA scores of the tumor intermediate state and the CD8+ T exhausted state in the TCGA-SKCM cohort (intermediate: *R* = 0.46, *p* < 2.2e-16; exhausted: *R* = 0.62, *p* < 2.2e-16) (**Figure 7E**). Intermediate tumor cells and exhausted CD8+ T cells were both significantly enriched in immunotherapy untreated samples compared to immunotherapy-resistant samples in the scRNA-seq dataset (**Figure 7F**, chi-squared test, intermediate *p* = 1.41e-64, exhausted *p* = 0.00334). At the same time, we observed that the shared ligands between the two cell states were generally expressed at higher levels in immunotherapy untreated samples (**Figures 7G**). Moreover, the correlation between the tumor intermediate state and the CD8+ T exhausted state was significant in the anti-CTLA4 untreated group (*R* = 0.48, *p* = 0.016), and was not found in the treatment-resistant group (**Figure 7H**). Additionally, responders had significantly higher CD8+ T exhausted state scores compared to non-responders during anti-PDCD1 treatment (Wilcoxon rank-sum test, *p* = 0.012) (**Figure 7I**). In summary, the tumor intermediate state and the CD8+ T exhausted state were synergistically associated with favorable prognosis and both reduced in immunotherapy-resistant samples.

### Genomic Mutations and Immune-Related Characteristics of Risk Groups

**Figure 8:**
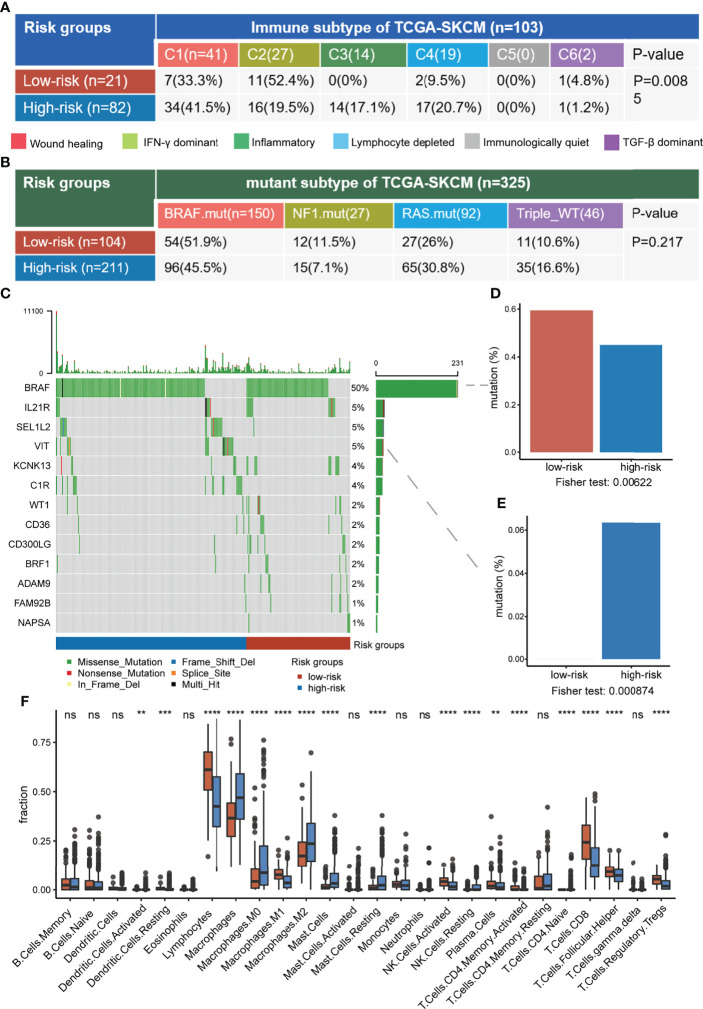
Comprehensive characterization of risk groups. **(A, B)** The association between risk groups with immune subtypes **(A)** and TCGA mutant subtypes **(B)** in the TCGA-SKCM cohort. **(C)** The oncoplot of selected genes with significantly different mutation fractions between risk groups. **(D, E)** The difference in mutation fraction of *BRAF* **(D)** and *VIT* **(E)** between risk groups. **(F)** The difference in immune infiltration between risk groups. ns, non-significant, * denoted p <0.05, ** denoted p <0.01, *** denoted p <0.001, and **** denoted p <0.0001.

According to the above survival curve analysis, samples were classified into high- and low-risk groups in the TCGA-SKCM cohort, where the low-risk group included the samples with higher tumor intermediate state and CD8+ T exhausted state scores. Then, the association between risk groups and other defined subtypes was investigated. For immune subtypes defined by immune signatures, the C2-interferon-gamma dominant subtype was particularly dominant in the low-risk group, while the high-risk group was enriched in C1-wound healing and C4-lymphocyte depleted subtypes (**Figure 8A**). The interplay of risk groups with the previously defined mutation-based molecular subtypes was also assessed. It was found that risk groups spanned across molecular subtypes and had no substantial heterogeneity in the distribution of molecular subtypes (**Figure 8B**). Subsequently, the prognostic analyses of immune and molecular subtypes showed that the *BRAF* mutated molecular subtype indicated more favorable survival than the other three groups, while the immune subtype was not a good indicator of survival (**Figure S6**).

We further investigated the differences in genomic and immune-related features between low-risk and high-risk groups. *BRAF* mutated frequently in the samples and it had a higher mutation fraction in the low-risk group compared to the high-risk overall (59.4% versus 45%, respectively, *p* = 0.006), consistent with the *BRAF* mutant subtype indicating longer survival (**Figures 8C,D**). Compared with the low-risk group, *VIT* associated with cell adhesion was mutated only in the high-risk group (6.3% versus 0%, respectively, *p* = 0.0009) (**Figure 8E**). In terms of immune infiltration, the low-risk group had obvious higher infiltration of antitumor immune-associated lymphocytes, such as CD8+ T, CD4+ T, and activated NK cells (**Figure 8F**). In conclusion, the low-risk group had a higher *BRAF* mutation fraction and stronger antitumor immune responses, suggesting that risk groups may comprehensively reflect both tumor genomic information and anti-tumor immune microenvironment.

## Discussion

In the present study, we deciphered the crosstalk propensity between the tumor intermediate state and the CD8+ T exhausted state by public scRNA-seq data. The tumor intermediate state was a transitional state from a melanocytic state to a mesenchymal-like state as previous research reported (3). The melanocytic state highly expressed pigmentation-associated genes (**Figure 1C**), which are documented to be recognized by T cells and thus contribute at least in part to the highly immunogenic nature of melanoma (51, 52). The absence of interferon-gamma response mediated by the antigen processing machinery in tumor cells may result in their failure to be recognized by the immune system (53, 54). The intersection of the MITF pathway and the interferon-gamma pathway was observed in the tumor intermediate state (**Figures 1A**, **3A**), which promoted tumor cells to be recognized by CD8+ T cells and might be the cause of the crosstalk propensity. In addition, the melanocytic state and the mesenchymal-like state were associated with resistance to target therapy (9–12), and the transition from a melanocytic state to a mesenchymal-like state was considered as an alternative route for acquiring drug resistance (10). The tumor intermediate state and the CD8+ T exhausted state were both associated with improved survival and both reduced in immunotherapy-resistant samples (**Figure 7**). Those results implying the transition of other tumor cell states to an intermediate state combined with immunotherapy may be a new therapeutic strategy.

Some shortcomings and prospects of this study should be addressed. A crosstalk propensity analysis on the association between CCC and cell state was performed due to the lack of sequencing data from physically interacting cells, which can improve the reliability (48). Currently, a few methods that rely on spatial transcriptome data are also emerging (55), and integrating physical contact-dependent and chemical signal-dependent CCCs will be a trend for future methods and algorithms. This study was completely based on public databases, and some of the key genes or results in this study need to be externally validated by further experiments, such as showing the direct proximity of crosstalk by immunohistochemistry.

In particular, there are many interesting details or challenges in crosstalk propensity analysis. We applied the NicheNet method to predict ligand activity for each signal receiver cell. Although the ligands we analyzed were expressed in tumor cells or CD8+ T cells, the effect of the ligands also expressed by other cells in the tumor microenvironment on the signal receiver cells cannot be excluded. However, at present, how to accurately decompose the ligand signal source of a receiver cell is still a problem to be solved, including the contribution of individual signal sender cells, and the signal transduction ability of individual receptors can interact with that ligand. One of the key questions is whether cell state-related ligands could be explained by the specific expression of receptors in a certain cell state influencing the ligand ligation and/or ligand engagement on cells promoting the state transition. In other words, whether CCC initiates cell state transition or cell state transition influences CCC, or whether these two cellular behaviors complement each other is difficult to answer in this study.

In addition, the effect of the tumor intermediate state and the CD8+ T exhausted state on patients does not directly represent the effect of CCC. However, dissociating CCC between different cell subpopulations from bulk expression profiles is a challenge. A deconvolution algorithm has been performed to obtain the expression profiles of tumor cells and stromal cells, which were further used to calculate the intensity of ligand–receptor interactions (56). This intensity is only the relative intensity of interactions with different modes of interaction (endocrine and exocrine), rather than absolute.

## Data Availability Statement

All of the data we used in this study were publicly available. The names of the repository/repositories and accession number(s) can be found in the “*Materials and Methods*” section.

## Author Contributions

XLi, YX, and YPZ contributed to the conception and design of the study. XLiang, ZDJ, GML, YZ, and WL collected the data. JLZ, MY, and HTY contributed to the data analysis. JLZ wrote the first draft of the manuscript. JLZ, MY, LWX and HTY revised the manuscript. All authors read and approved the final manuscript.

## Funding

This work was supported by the National Key R&D Program of China (Grant No. 2018YFC2000100), the National Natural Science Foundation of China (Grant Nos. 62172131, 32070673, and 31871336), the Heilongjiang Provincial Natural Science Foundation (Grant Nos. YQ2019C012 and YQ2021C026), the Heilongjiang Postdoctoral Foundation (Grant No. LBH-Q18099), Program for Young Scholars with Creative Talents in Heilongjiang Province (UNPYSCT-2017059), and the Heilongjiang Touyan Innovation Team Program.

## Conflict of Interest

The authors declare that the research was conducted in the absence of any commercial or financial relationships that could be construed as a potential conflict of interest.

## Publisher’s Note

All claims expressed in this article are solely those of the authors and do not necessarily represent those of their affiliated organizations, or those of the publisher, the editors and the reviewers. Any product that may be evaluated in this article, or claim that may be made by its manufacturer, is not guaranteed or endorsed by the publisher.

## Supplementary Material

Supplementary Table 1 TF regulons and functional signatures or markers for annotating tumor cells and CD8+ T cell states.

Supplementary Table 2 Ligand-receptor resources.

## References

[1] Alexandrov LB Nik-Zainal S Wedge DC Aparicio SA Behjati S Biankin AV. Signatures of Mutational Processes in Human Cancer. Nature (2013) 500(7463):415–21. doi: 10.1038/nature12477 PMC3776390 23945592

[2] Grzywa TM Paskal W Wlodarski PK. Intratumor and Intertumor Heterogeneity in Melanoma. Transl Oncol (2017) 10(6):956–75. doi: 10.1016/j.tranon.2017.09.007 PMC5671412 29078205

[3] Wouters J Kalender-Atak Z Minnoye L Spanier KI De Waegeneer M Bravo Gonzalez-Blas C. Robust Gene Expression Programs Underlie Recurrent Cell States and Phenotype Switching in Melanoma. Nat Cell Biol (2020) 22(8):986–98. doi: 10.1038/s41556-020-0547-3 32753671

[4] Tirosh I Izar B Prakadan SM Wadsworth MH Treacy D Trombetta JJ. Garraway: Dissecting the Multicellular Ecosystem of Metastatic Melanoma by Single-Cell RNA-Seq. Science (2016) 352(6282):189–96. doi: 10.1126/science.aad0501 PMC4944528 27124452

[5] Sade-Feldman M Yizhak K Bjorgaard SL Ray JP de Boer CG Jenkins RW. Defining T Cell States Associated With Response to Checkpoint Immunotherapy in Melanoma. Cell (2018) 175(4):998–1013.e20. doi: 10.1016/j.cell.2018.10.038 30388456 PMC6641984

[6] Carmona SJ Siddiqui I Bilous M Held W Gfeller D. Deciphering the Transcriptomic Landscape of Tumor-Infiltrating CD8 Lymphocytes in B16 Melanoma Tumors With Single-Cell RNA-Seq. Oncoimmunology (2020) 9(1):1737369. doi: 10.1080/2162402X.2020.1737369 32313720 PMC7153840

[7] Rambow F Marine JC Goding CR. Melanoma Plasticity and Phenotypic Diversity: Therapeutic Barriers and Opportunities. Genes Dev (2019) 33(19-20):1295–318. doi: 10.1101/gad.329771.119 PMC6771388 31575676

[8] Li H van der Leun AM Yofe I Lubling Y Gelbard-Solodkin D van Akkooi ACJ. Dysfunctional CD8 T Cells Form a Proliferative, Dynamically Regulated Compartment Within Human Melanoma. Cell (2019) 176(4):775–789.e18. doi: 10.1016/j.cell.2018.11.043 30595452 PMC7253294

[9] Konieczkowski DJ Johannessen CM Abudayyeh O Kim JW Cooper ZA Piris A. A Melanoma Cell State Distinction Influences Sensitivity to MAPK Pathway Inhibitors. Cancer Discovery (2014) 4(7):816–27. doi: 10.1158/2159-8290.CD-13-0424 PMC4154497 24771846

[10] Kemper K de Goeje PL Peeper DS van Amerongen R. Phenotype Switching: Tumor Cell Plasticity as a Resistance Mechanism and Target for Therapy. Cancer Res (2014) 74(21):5937–41. doi: 10.1158/0008-5472.CAN-14-1174 25320006

[11] Titz B Lomova A Le A Hugo W Kong X Ten Hoeve J. JUN Dependency in Distinct Early and Late BRAF Inhibition Adaptation States of Melanoma. Cell Discov (2016) 2:16028. doi: 10.1038/celldisc.2016.28 27648299 PMC5012007

[12] Shaffer SM Dunagin MC Torborg SR Torre EA Emert B Krepler C. Rare Cell Variability and Drug-Induced Reprogramming as a Mode of Cancer Drug Resistance. Nature (2017) 546(7658):431–5. doi: 10.1038/nature22794 PMC5542814 28607484

[13] Rambow F Rogiers A Marin-Bejar O Aibar S Femel J Dewaele M. Toward Minimal Residual Disease-Directed Therapy in Melanoma. Cell (2018) 174(4):843–855.e19. doi: 10.1016/j.cell.2018.06.025 30017245

[14] Cohen M Giladi A Gorki AD Solodkin DG Zada M Hladik A. Lung Single-Cell Signaling Interaction Map Reveals Basophil Role in Macrophage Imprinting. Cell (2018) 175(4):1031–1044.e18. doi: 10.1016/j.cell.2018.09.009 30318149

[15] Boisset JC Vivie J Grun D Muraro MJ Lyubimova A van Oudenaarden A. Mapping the Physical Network of Cellular Interactions. Nat Methods (2018) 15(7):547–53. doi: 10.1038/s41592-018-0009-z 29786092

[16] Qiao W Wang W Laurenti E Turinsky AL Wodak SJ Bader GD. Intercellular Network Structure and Regulatory Motifs in the Human Hematopoietic System. Mol Syst Biol (2014) 10:741. doi: 10.15252/msb.20145141 25028490 PMC4299490

[17] Paik DT Tian L Lee J Sayed N Chen IY Rhee S. Large-Scale Single-Cell RNA-Seq Reveals Molecular Signatures of Heterogeneous Populations of Human Induced Pluripotent Stem Cell-Derived Endothelial Cells. Circ Res (2018) 123(4):443–50. doi: 10.1161/CIRCRESAHA.118.312913 PMC6202208 29986945

[18] Li G Tian L Goodyer W Kort EJ Buikema JW Xu A. Single Cell Expression Analysis Reveals Anatomical and Cell Cycle-Dependent Transcriptional Shifts During Heart Development. Development (2019) 146(12):dev173476. doi: 10.1242/dev.173476 31142541 PMC6602356

[19] Kumar MP Du J Lagoudas G Jiao Y Sawyer A Drummond DC. Analysis of Single-Cell RNA-Seq Identifies Cell-Cell Communication Associated With Tumor Characteristics. Cell Rep (2018) 25(6):1458–1468.e4. doi: 10.1016/j.celrep.2018.10.047 30404002 PMC7009724

[20] Martin JC Chang C Boschetti G Ungaro R Giri M Grout JA. Single-Cell Analysis of Crohn's Disease Lesions Identifies a Pathogenic Cellular Module Associated With Resistance to Anti-TNF Therapy. Cell (2019) 178(6):1493–1508.20. doi: 10.1016/j.cell.2019.08.008 31474370 PMC7060942

[21] Wei F Zhong S Ma Z Kong H Medvec A Ahmed R. Strength of PD-1 Signaling Differentially Affects T-Cell Effector Functions. Proc Natl Acad Sci U.S.A. (2013) 110(27):E2480–9. doi: 10.1073/pnas.1305394110 PMC3703988 23610399

[22] Juneja VR McGuire KA Manguso RT LaFleur MW Collins N Haining WN. PD-L1 on Tumor Cells is Sufficient for Immune Evasion in Immunogenic Tumors and Inhibits CD8 T Cell Cytotoxicity. J Exp Med (2017) 214(4):895–904. doi: 10.1084/jem.20160801 28302645 PMC5379970

[23] Chen L Diao L Yang Y Yi X Rodriguez BL Li Y. CD38-Mediated Immunosuppression as a Mechanism of Tumor Cell Escape From PD-1/PD-L1 Blockade. Cancer Discovery (2018) 8(9):1156–75. doi: 10.1158/2159-8290.CD-17-1033 PMC6205194 30012853

[24] Bagati A Kumar S Jiang P Pyrdol J Zou AE Godicelj A. Integrin Alphavbeta6-TGFbeta-SOX4 Pathway Drives Immune Evasion in Triple-Negative Breast Cancer. Cancer Cell (2021) 39(1):54–67.e9. doi: 10.1016/j.ccell.2020.12.001 33385331 PMC7855651

[25] van der Leun AM Thommen DS Schumacher TN. CD8(+) T Cell States in Human Cancer: Insights From Single-Cell Analysis. Nat Rev Cancer (2020) 20(4):218–32. doi: 10.1038/s41568-019-0235-4 PMC7115982 32024970

[26] Fu T Dai LJ Wu SY Xiao Y Ma D Jiang YZ. Spatial Architecture of the Immune Microenvironment Orchestrates Tumor Immunity and Therapeutic Response. J Hematol Oncol (2021) 14(1):98. doi: 10.1186/s13045-021-01103-4 34172088 PMC8234625

[27] Gide TN Silva IP Quek C Ahmed T Menzies AM Carlino MS. Close Proximity of Immune and Tumor Cells Underlies Response to Anti-PD-1 Based Therapies in Metastatic Melanoma Patients. Oncoimmunology (2020) 9(1):1659093. doi: 10.1080/2162402X.2019.1659093 32002281 PMC6959449

[28] Hao Y Hao S Andersen-Nissen E Mauck WM Zheng S Butler A. Integrated Analysis of Multimodal Single-Cell Data. bioRxiv (2020) 2020:10.12.335331. doi: 10.1101/2020.10.12.335331 PMC8238499 34062119

[29] Thorsson V Gibbs DL Brown SD Wolf D Bortone DS Ou Yang TH. The Immune Landscape of Cancer. Immunity (2018) 48(4):812–830.e14. doi: 10.1016/j.immuni.2018.03.023 29628290 PMC5982584

[30] Thrane K Eriksson H Maaskola J Hansson J Lundeberg J. Spatially Resolved Transcriptomics Enables Dissection of Genetic Heterogeneity in Stage III Cutaneous Malignant Melanoma. Cancer Res (2018) 78(20):5970–9. doi: 10.1158/0008-5472.CAN-18-0747 30154148

[31] Aibar S Gonzalez-Blas CB Moerman T Huynh-Thu VA Imrichova H Hulselmans G. SCENIC: Single-Cell Regulatory Network Inference and Clustering. Nat Methods (2017) 14(11):1083–6. doi: 10.1038/nmeth.4463 PMC5937676 28991892

[32] Gu Z Eils R Schlesner M. Complex Heatmaps Reveal Patterns and Correlations in Multidimensional Genomic Data. Bioinformatics (2016) 32(18):2847–9. doi: 10.1093/bioinformatics/btw313 27207943

[33] Yuan H Yan M Zhang G Liu W Deng C Liao G. CancerSEA: A Cancer Single-Cell State Atlas. Nucleic Acids Res (2019) 47(D1):D900–8. doi: 10.1093/nar/gky939 PMC6324047 30329142

[34] Trapnell C Cacchiarelli D Grimsby J Pokharel P Li S Morse M. The Dynamics and Regulators of Cell Fate Decisions are Revealed by Pseudotemporal Ordering of Single Cells. Nat Biotechnol (2014) 32(4):381–6. doi: 10.1038/nbt.2859 PMC4122333 24658644

[35] Ramilowski JA Goldberg T Harshbarger J Kloppmann E Lizio M Satagopam VP. A Draft Network of Ligand-Receptor-Mediated Multicellular Signalling in Human. Nat Commun (2015) 6:7866. doi: 10.1038/ncomms8866 26198319 PMC4525178

[36] Wang Y Wang R Zhang S Song S Jiang C Han G. iTALK: An R Package to Characterize and Illustrate Intercellular Communication. bioRxiv (2019), 507871. doi: 10.1101/507871

[37] Cabello-Aguilar S Alame M Kon-Sun-Tack F Fau C Lacroix M Colinge J. SingleCellSignalR: Inference of Intercellular Networks From Single-Cell Transcriptomics. Nucleic Acids Res (2020) 48(10):e55. doi: 10.1093/nar/gkaa183 32196115 PMC7261168

[38] Shao X Liao J Li C Lu X Cheng J Fan X. CellTalkDB: A Manually Curated Database of Ligand-Receptor Interactions in Humans and Mice. Brief Bioinform (2020) 22(4):bbaa269. doi: 10.1093/bib/bbaa269 33147626

[39] Ren X Zhong G Zhang Q Zhang L Sun Y Zhang Z. Reconstruction of Cell Spatial Organization From Single-Cell RNA Sequencing Data Based on Ligand-Receptor Mediated Self-Assembly. Cell Res (2020) 30(9):763–78. doi: 10.1038/s41422-020-0353-2 PMC7608415 32541867

[40] Zhou Y Zhou B Pache L Chang M Khodabakhshi AH Tanaseichuk O. Metascape Provides a Biologist-Oriented Resource for the Analysis of Systems-Level Datasets. Nat Commun (2019) 10(1):1523. doi: 10.1038/s41467-019-09234-6 30944313 PMC6447622

[41] Browaeys R Saelens W Saeys Y. NicheNet: Modeling Intercellular Communication by Linking Ligands to Target Genes. Nat Methods (2020) 17(2):159–62. doi: 10.1038/s41592-019-0667-5 31819264

[42] Cheng J Zhang J Wu Z Sun X. Inferring Microenvironmental Regulation of Gene Expression From Single-Cell RNA Sequencing Data Using Scmlnet With an Application to COVID-19. Brief Bioinform (2021) 22(2):988–1005. doi: 10.1093/bib/bbaa327 33341869 PMC7799217

[43] Subramanian A Tamayo P Mootha VK Mukherjee S Ebert BL Gillette MA. Gene Set Enrichment Analysis: A Knowledge-Based Approach for Interpreting Genome-Wide Expression Profiles. Proc Natl Acad Sci U.S.A. (2005) 102(43):15545–50. doi: 10.1073/pnas.0506580102 PMC1239896 16199517

[44] Creighton CJ Li X Landis M Dixon JM Neumeister VM Sjolund A. Residual Breast Cancers After Conventional Therapy Display Mesenchymal as Well as Tumor-Initiating Features. Proc Natl Acad Sci U.S.A. (2009) 106(33):13820–5. doi: 10.1073/pnas.0905718106 PMC2720409 19666588

[45] Broutin S Ameur N Lacroix L Robert T Petit B Oumata N. Identification of Soluble Candidate Biomarkers of Therapeutic Response to Sunitinib in Medullary Thyroid Carcinoma in Preclinical Models. Clin Cancer Res (2011) 17(7):2044–54. doi: 10.1158/1078-0432.CCR-10-2041 21325074

[46] Barry M Bleackley RC. Cytotoxic T Lymphocytes: All Roads Lead to Death. Nat Rev Immunol (2002) 2(6):401–9. doi: 10.1038/nri819 12093006

[47] Kinslechner K Schutz B Pistek M Rapolter P Weitzenbock HP Hundsberger H. Loss of SR-BI Down-Regulates MITF and Suppresses Extracellular Vesicle Release in Human Melanoma. Int J Mol Sci (2019) 20(5):1063. doi: 10.3390/ijms20051063 PMC6429474 30823658

[48] Shao X Lu X Liao J Chen H Fan X. New Avenues for Systematically Inferring Cell-Cell Communication: Through Single-Cell Transcriptomics Data. Protein Cell (2020) 11(12):866–80. doi: 10.1007/s13238-020-00727-5 PMC7719148 32435978

[49] Nirmal AJ Maliga Z Vallius T Quattrochi B Chen AA Jacobson CA. The Spatial Landscape of Progression and Immunoediting in Primary Melanoma at Single Cell Resolution. Cancer Discovery (2022) 12(6):1518–1541. doi: 10.1158/2159-8290.CD-21-1357 PMC9167783 35404441

[50] Iwanaga R Truong BT Hsu JY Lambert KA Vyas R Orlicky D. Loss of Prdm1a Accelerates Melanoma Onset and Progression. Mol Carcinog (2020) 59(9):1052–63. doi: 10.1002/mc.23236 PMC7864383 32562448

[51] Jager E Ringhoffer M Karbach J Arand M Oesch F Knuth A. Inverse Relationship of Melanocyte Differentiation Antigen Expression in Melanoma Tissues and CD8+ Cytotoxic-T-Cell Responses: Evidence for Immunoselection of Antigen-Loss Variants *In Vivo* . Int J Cancer (1996) 66(4):470–6. doi: 10.1002/(SICI)1097-0215(19960516)66:4<470::AID-IJC10>3.0.CO;2-C 8635862

[52] Slingluff CL Jr. Colella TA Thompson L Graham DD Skipper JC Caldwell J. Melanomas With Concordant Loss of Multiple Melanocytic Differentiation Proteins: Immune Escape That may be Overcome by Targeting Unique or Undefined Antigens. Cancer Immunol Immunother (2000) 48(12):661–72. doi: 10.1007/s002620050015 PMC11037162 10752474

[53] Bai X Fisher DE Flaherty KT. Cell-State Dynamics and Therapeutic Resistance in Melanoma From the Perspective of MITF and IFNgamma Pathways. Nat Rev Clin Oncol (2019) 16(9):549–62. doi: 10.1038/s41571-019-0204-6 PMC7185899 30967646

[54] Grasso CS Tsoi J Onyshchenko M Abril-Rodriguez G Ross-Macdonald P Wind-Rotolo M. Conserved Interferon-Gamma Signaling Drives Clinical Response to Immune Checkpoint Blockade Therapy in Melanoma. Cancer Cell (2020) 38(4):500–515.e3. doi: 10.1016/j.ccell.2020.08.005 32916126 PMC7872287

[55] Cang Z Nie Q. Inferring Spatial and Signaling Relationships Between Cells From Single Cell Transcriptomic Data. Nat Commun (2020) 11(1):2084. doi: 10.1038/s41467-020-15968-5 32350282 PMC7190659

[56] Ghoshdastider U Rohatgi N Mojtabavi Naeini M Baruah P Revkov E Guo YA. Pan-Cancer Analysis of Ligand-Receptor Cross-Talk in the Tumor Microenvironment. Cancer Res (2021) 81(7):1802–12. doi: 10.1158/0008-5472.CAN-20-2352 33547160

