# Supplementary material for: Single-Cell Transcriptomic Analysis Reveals the Crosstalk Propensity Between the Tumor Intermediate State and the CD8+ T Exhausted State to be Associated with Clinical Benefits in Melanoma
Source: Front Immunol. 2022 Jul 12;13:766852. doi: 10.3389/fimmu.2022.766852 (PMC9314667; doi:10.3389/fimmu.2022.766852)
Supplement: Supplementary file 3 [file DataSheet_1.docx]

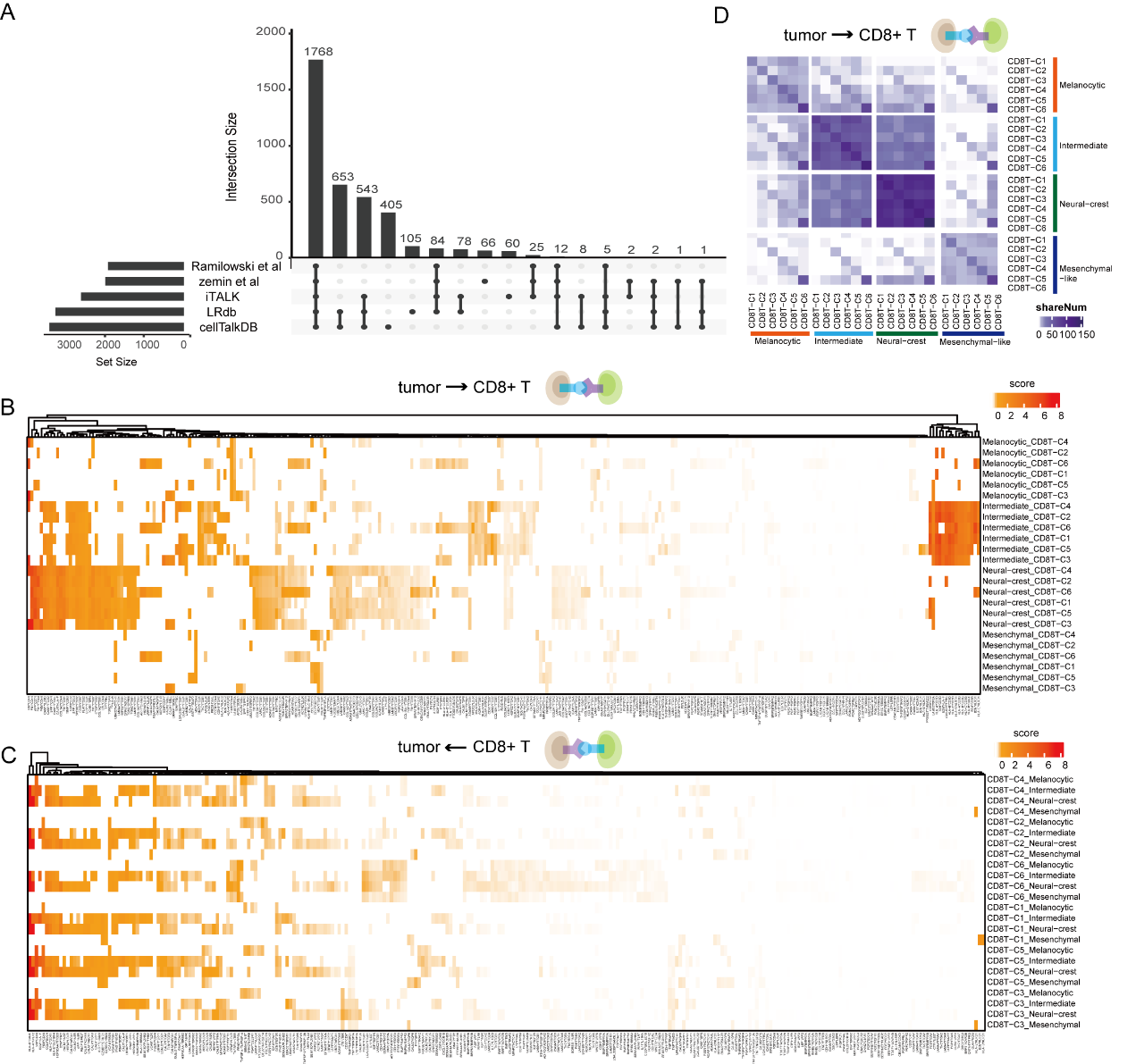


**Supplementary Fig.1. Ligand-receptor pairs integration and interactions inference. (A)** The intersection of five ligand-receptor resources. **(B-C)** Overview of ligand-receptor interactions between tumor cells and CD8+ T cells when tumor cells as senders **(B)** and CD8+ T cells as senders **(C)**. **(D)** The shared ligand-receptor intersections between tumor intermediate state and neural-crest-like state with CD8+ T cells when tumor cells as senders.

**
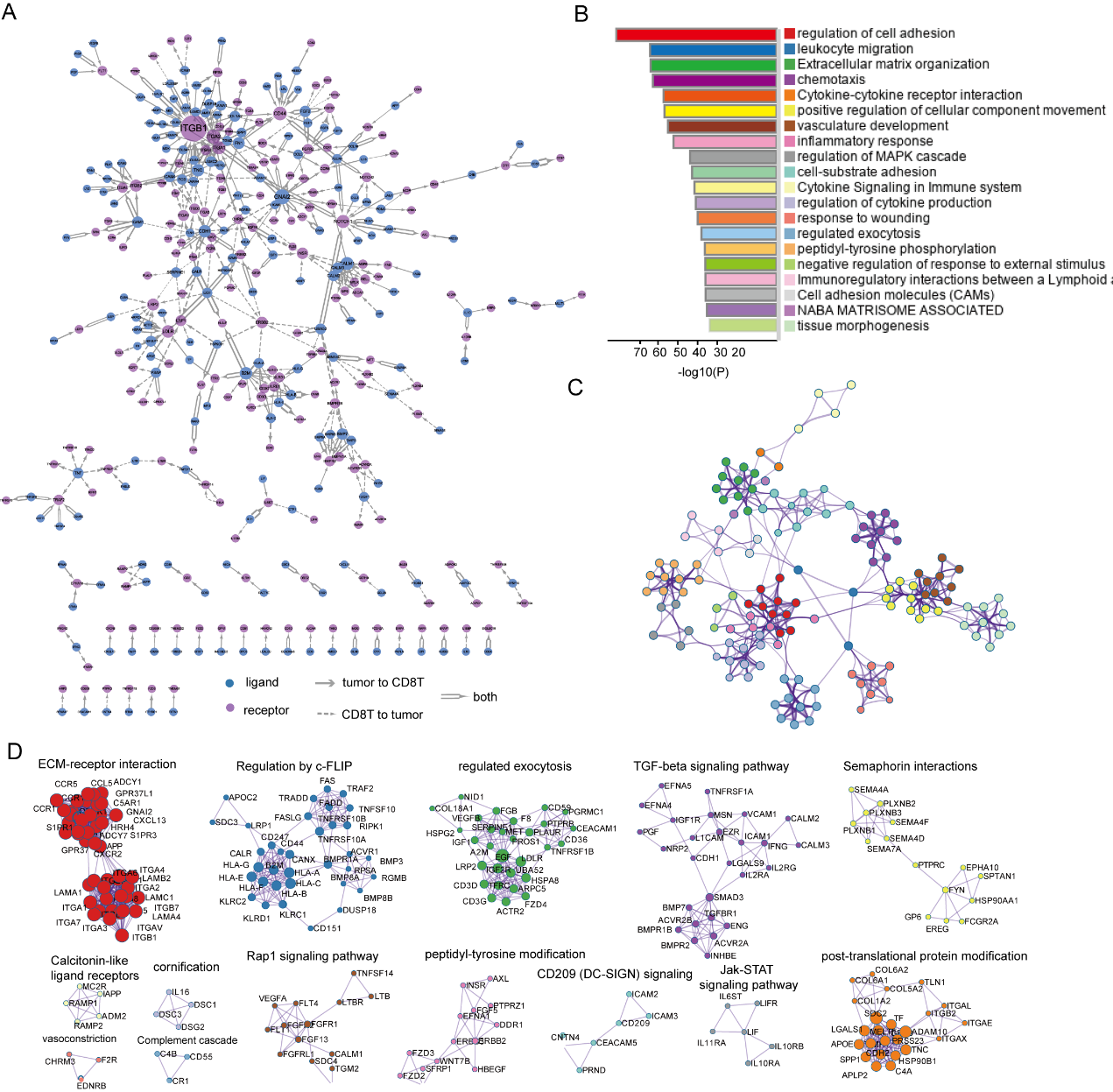
**

**Supplementary Fig. 2. Ligand-receptor network and function enrichment analysis. (A)** Overview of integrated ligand-receptor interaction network between tumor cells and CD8+ T cells. Node size represented the degree of the node. The line type labeled by the directions of signal transduction and thickness showed interaction frequency between the tumor cells and the CD8+ T cells. **(B)** Bar graph for viewing top non-redundant enrichment results of genes included in the ligand-receptor network. **(C)** Enrichment network visualization showing the intra-cluster and inter-cluster similarities of enriched terms, up to ten terms per cluster. **(D)** MCODE complexes colored by their identities and their functional labels were shown above.

**
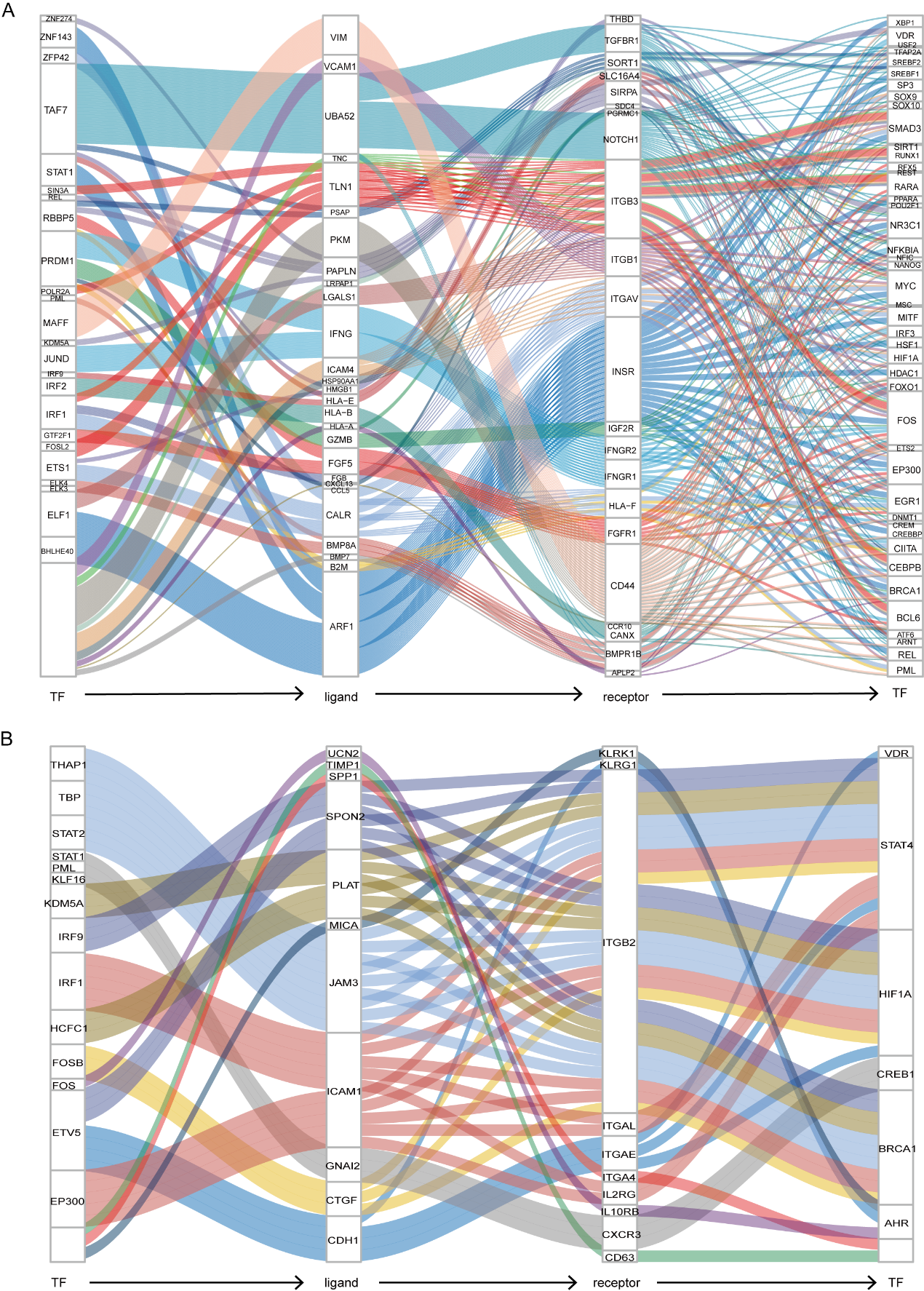
**

**Supplementary Fig.****3. Inter/intra-cellular signal transduction network** **between tumor intermediate state and** **CD8+ T exhausted state.** Sankey plot of inter/intra-cellular signal transduction network when CD8+ T exhausted state as senders **(A)** and tumor intermediate state as senders **(B)**. The sine is colored by ligands.

**
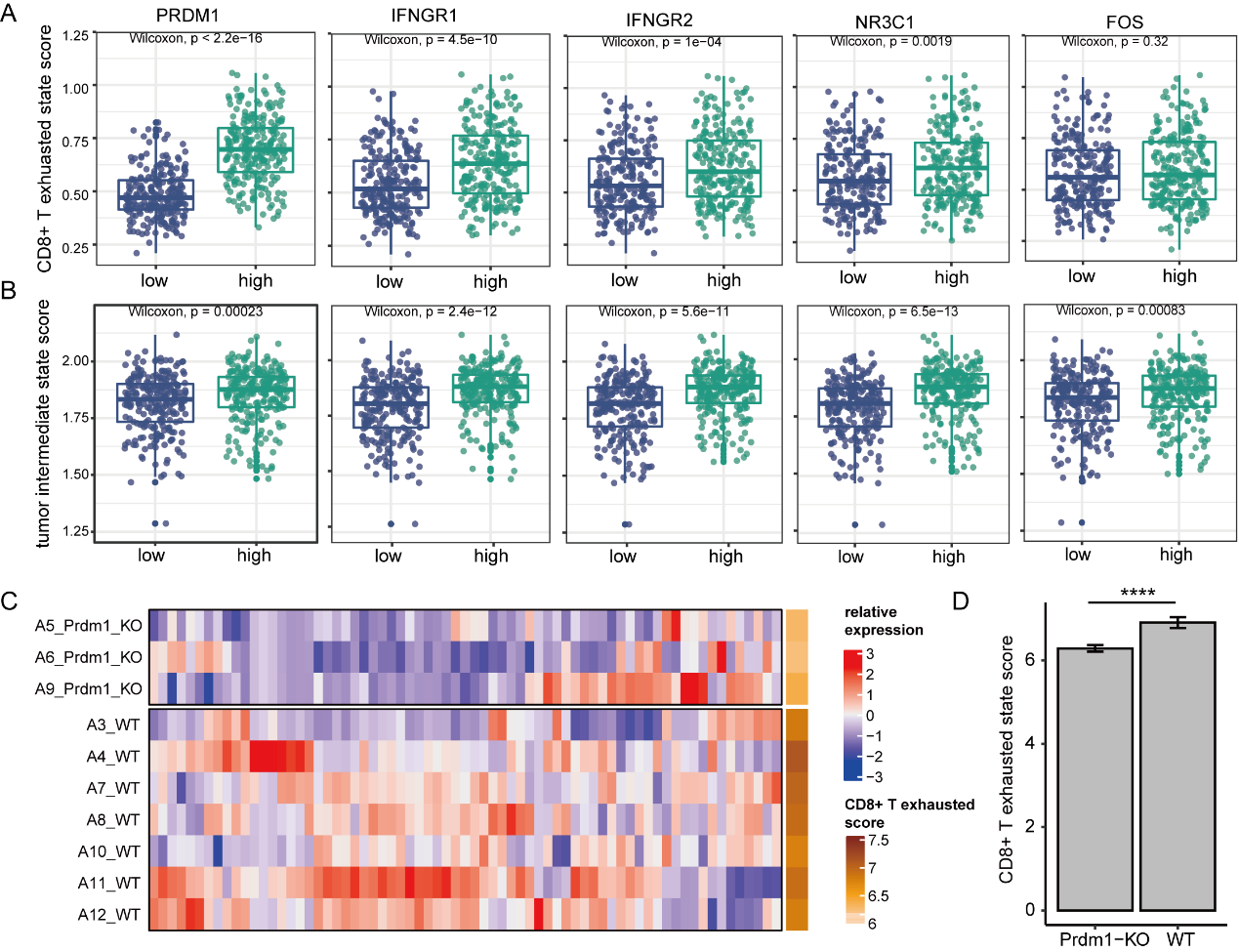
**

**Supplementary Fig.4.** **Exploration of the association between TF-ligand-receptor-TF molecules and cell states. (A-B)** In the TCGA-SKCM cohort, the comparison of CD8+ T exhausted state ssGSEA score**(A)** and tumor intermediate state ssGSEA score**(B)** between high- and low-expressed groups defined by the median expression level of *PRDM1*, *IFNGR1*, *IFNGR2*, *NR3C1*, and *FOS* respectively. **(C)** The comparison of CD8+ T exhausted specific signature expression levels in CD8+ tumor-infiltrating lymphocytes between *Prdm1* cKO mice (n=3) and wild type (n=7). (D) The ssGSEA scores of CD8+ T exhausted state in CD8+ tumor-infiltrating lymphocytes from *Prdm1* cKO mice (n=3) and wild type (n=7). mean + s.e.m is shown. p = 6.281e-05, two-sided t-test.

**
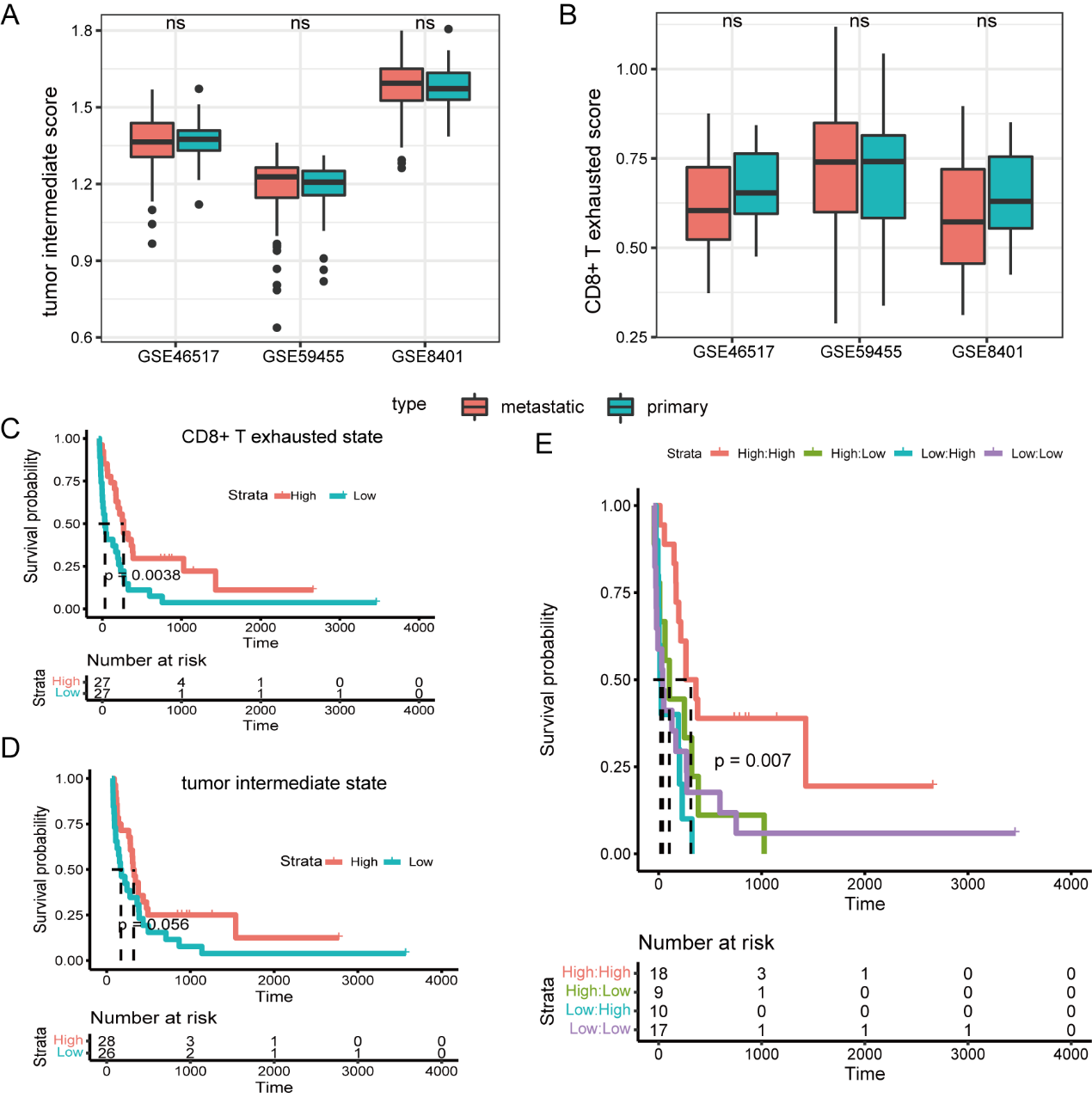
**

**Supplementary Fig.5.** **Association between clinical phenotype and** **tumor intermediate state and** **CD8+ T exhausted state. (A-B)** The difference in tumor intermediate state ssGSEA score **(A)** and CD8+ T exhausted state ssGSEA score **(B)** between primary and metastatic samples in three melanoma cohorts. **(C-E)** Kaplan–Meier survival curves of overall survival by CD8+ T exhausted state ssGSEA score **(C)**, tumor intermediate state ssGSEA score **(D)**, and the combination of the above two variables **(E)** in the GSE22153 cohort.


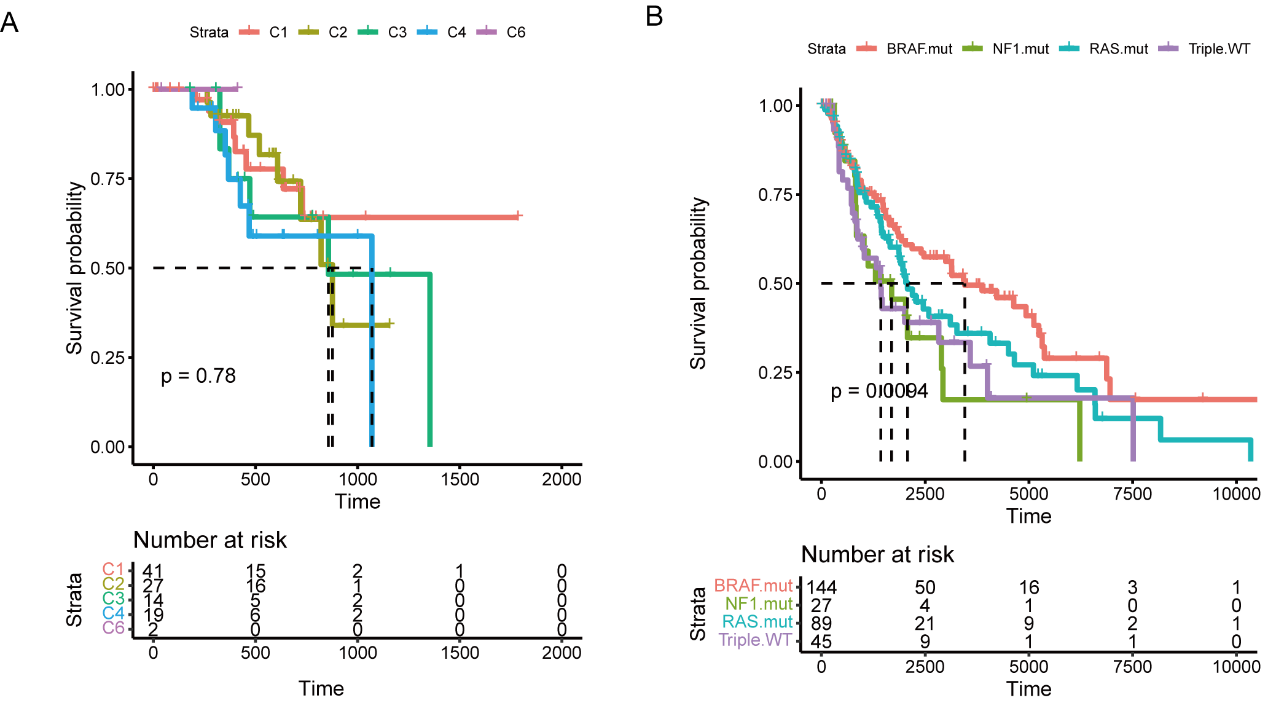


**Supplementary Fig.6. Survival curve analysis of overall survival for immune subtypes and mutant subtypes.** The association between over survival and immune subtypes shown in **(A)** and mutant subtypes shown in **(B)**.
